# Colorectal cancer screening using the faecal occult blood test (FOBt): a survey of GP attitudes and practices in the UK

**DOI:** 10.1186/1471-2296-11-20

**Published:** 2010-03-09

**Authors:** Sarah Damery, Sue Clifford, Sue Wilson

**Affiliations:** 1 Primary Care Clinical Sciences, University of Birmingham, Edgbaston, Birmingham, B15 2TT, UK

## Abstract

**Background:**

Colorectal cancer (CRC) is the third most common cancer in the UK. Five-year survival rates are less than 50%, largely because of late diagnosis. Screening using faecal occult blood tests (FOBt) can detect bowel cancer at an earlier stage than symptomatic presentation, and has the potential to significantly decrease colorectal cancer mortality. However, uptake of screening is currently low, despite the introduction of the NHS Bowel Cancer Screening Programme (NHSBCSP), and it has been suggested that GP recommendations of screening can improve patient compliance. GP recommendation of CRC screening is argued to be affected by attitudes towards it, along with perceptions of its efficacy.

**Methods:**

This paper presents the findings of a cross-sectional postal survey of GPs in the UK which aimed to investigate GPs' attitudes in relation to colorectal cancer screening and the use of FOBt in routine practice. An 'attitude' score was calculated, and binary logistic regression used to evaluate the association of socio-demographic and general practice attributes with attitudes towards CRC screening and FOBt.

**Results:**

Of 3,191 GPs surveyed, 960 returned usable responses (response rate 30.7%). Positive attitudes were associated with personal experience of CRC screening and Asian or Asian British ethnicity. GPs from practices located in more deprived locations were also more likely to have positive attitudes towards FOBt and its recommendation to patients.

**Conclusions:**

The success of population-based screening for CRC will largely be determined by GP attitudes and support, particularly with regard to FOBt. Previous research has implied that South Asian GPs are more likely to have negative attitudes towards FOBt screening, however, our research suggests that this is not a group requiring targeted interventions to increase their support for the NHSBCSP. Of the available CRC screening tests, GPs perceived FOBt to be the most appropriate for population-based screening.

## Background

Colorectal cancer (CRC) is the third most common cancer, and the second leading cause of cancer death in the UK, with 35,000 diagnoses and 16,000 deaths per year [1]. It incurs an annual National Health Service (NHS) expenditure of more than £300 million in surgical, adjuvant and palliative treatment [2]. Despite this, the five-year CRC survival rate is currently only 48% [3]; lower than in other European countries [4,5]. This poor survival rate is largely attributable to late diagnosis; therefore an effective means of improving CRC survival and reducing the burden of the disease to the NHS is to facilitate early diagnosis and treatment through CRC screening [1].

Biennial bowel cancer screening using the faecal occult blood test (FOBt) has been shown to have the potential to reduce mortality from bowel cancer by 16% [6]. FOBt screening in the asymptomatic average-risk population can detect bowel cancer at an earlier stage than would be the case through symptomatic presentation, increasing the potential effectiveness of treatment [7]. It also allows the identification of precursors to invasive disease which can be removed subsequently during colonoscopy to lessen the likelihood of CRC developing. In addition to improved survival, earlier diagnosis through CRC screening can contribute to improved quality of life and reduced NHS treatment costs [8]. However, if the potential morbidity and mortality benefits are to be realised, high levels of screening uptake and the continued adherence to regular screening must be achieved and maintained in the eligible population.

The NHS Bowel Cancer Screening Programme (NHSBCSP) was introduced in England in 2006 [9]. The programme aims to screen men and women aged between 60 and 74 years for CRC every two years using FOBt, and it is estimated that there would be 20,000 fewer deaths from CRC over the next 20 years if screening had an uptake of 60% [10]. However, current uptake is 52% [11], a rate which appears to be falling in rounds subsequent to the prevalent round [12]. Uptake is considerably lower than this in some population sub-groups, with men, those in younger age groups, those from the Indian sub-continent and people living in deprived areas least likely to participate [11,13]. Whilst General Practitioners (GPs) in the UK are not directly involved in administering bowel cancer screening, the national pilot study for the NHSBCSP demonstrated that GP attitudes to screening appear to be an important determinant of uptake [14]. GP involvement has been shown to improve compliance with CRC screening in general [15-17], and to increase uptake of FOBt in particular [18,19]. If the potential benefits of bowel cancer screening are to be realised, GPs must be actively engaged [20].

Studies undertaken internationally suggest that the beliefs and practices of GPs in relation to CRC screening and FOBt vary widely. The recommendation of CRC screening has been found to be associated with GP perceptions of screening and test efficacy [21]; the existence of guidelines underpinning clinical practice [22], and training [23]. Socio-demographic factors such as GP age, gender and ethnicity have been found to have mixed influences [14,23,24], as have GP practice attributes such as practice size, number of registered patients and practice location [22,24]. However, much of this existing research has focused on opportunistic rather than population-based screening [15], and due to differences in national healthcare systems and in the relative involvement of primary care in delivering CRC screening, studies undertaken outside of the UK may have limited applicability to the UK context.

This paper presents the findings of a cross-sectional postal survey of GPs in the UK which aimed to investigate GPs' attitudes in relation to colorectal cancer screening and the use of FOBt in routine practice.

## Methods

### Participants

A random sample of GPs (n = 31,358) was selected using the MidReC database (derived from the Prescription Pricing Division Database for England). The sample was stratified by practice size, and, by linking practice postcode to the Index of Multiple Deprivation (IMD) 2004 [25], by deprivation quartile. The IMD 2004 is a weighted area level aggregation of a number of 'domains' of deprivation (income, employment, health inequality, disability, education, skills and training, barriers to housing services, crime and the living environment). Lower IMD scores indicate more deprived areas, whereas higher scores are associated with less deprived locations. A 'small' practice was defined as one having three GPs or fewer (n = 7,987; 25.9%), and 'multiple' as having four GPs or more (n = 22,874; 74.1%). 'Affluent' practices were defined as those with an IMD 2004 rank of 16242 or higher (n = 17,654; 57.2%), and 'deprived' practices as those with an IMD 2004 rank of 16241 or lower (n = 13,207; 42.8%). Those with no IMD rank were excluded (n = 497; 1.6%). Combining these factors, all practices in the sample were stratified according to practice size and IMD quartile, and each was allocated to one of four groups: small/affluent (n = 5,295; 16.9%), small/deprived (n = 2,692; 8.6%), multiple/affluent (n = 12,359; 39.4%), or multiple/deprived (n = 10,515; 33.5%). 800 GPs were randomly selected from each of these four groups.

After excluding nine GPs who had recently been contacted as part of a medical student project on attitudes to CRC screening, self-completion surveys were sent to a total of 3,191 GPs between August and October 2007. This mailing assumed a conservative 40% response rate, to yield a sample size sufficient to determine the overall proportion of respondents with negative attitudes towards, and resistance to, FOBt based screening with 5% precision (95% confidence), based on a worst case scenario of 50% reporting such attitudes. Recipients wishing to participate were able to return the survey directly to the research team using an enclosed Freepost envelope. Non-respondents received one reminder. No incentive was offered for survey completion. Mailing was undertaken in October and November 2007, during the period where the National Bowel Screening Programme was being rolled out.

### Survey

The survey (Additional file 1) was a modified version of a postal questionnaire designed by the US National Cancer Institute (NCI) colorectal cancer screening team [26]. Prior to mailing, the modified survey was piloted using a sample of GPs within the Department of Primary Care, University of Birmingham to test ease of completion and to ensure comprehensibility. The survey included closed questions with categorical and Likert scale response options. Questions focused on the perceived effectiveness of cancer screening in general, and colorectal cancer screening in particular; factors influencing GP recommendation of CRC screening; perceived patient and system-related barriers to CRC screening, and current practice in relation to screening using FOBt for asymptomatic average-risk patients. Further questions gained information on socio-demographic characteristics of respondents (ethnicity, personal experience of CRC screening, affiliation with medical school) and GP practice attributes (practice setting, number of partners, list size, age profile of registered patients).

### Data analysis

Analysis focused on the socio-demographic characteristics and general practice attributes of respondents, and their relationship with stated attitudes towards the recommendation or otherwise of FOBt. A score was calculated based on the responses to three survey questions to represent a proxy measure of GP attitudes to FOBt screening (Table 1). Possible attitude scores ranged from a minimum of three to a maximum of nine. Responses were dichotomised between those with an attitude score of seven and above (representing a positive attitude), and those scoring six or lower (negative attitude). Binary logistic regression was used to calculate bivariate Odds Ratios to evaluate the association of socio-demographic and practice attributes with attitudes towards FOBt. Data were analysed using parametric or non-parametric tests for comparison of means and proportions as appropriate. This included analysis of differences between survey responders and non-responders where data on the relevant characteristics for non-responders (practice size, deprivation quartile, and ethnicity) could be obtained from the MidReC database. All data were analysed using SPSS (version 14.0).

**Table 1:** Calculation of 'attitude' score for each respondent as a proxy measure of attitudes to FOBt screening

Survey question	Score assigned for each response*§		
	**1**	**2**	**3**
How effective do you believe FOBt is in reducing cancer mortality in average risk patients aged 50 years and older?	Not effective	Somewhat effective	Very effective
How often do you recommend FOBt for your asymptomatic average-risk patients of the appropriate age?	Rarely/Never	Sometimes	Almost always
How appropriate do you think FOBt is for population-based colorectal cancer screening?	Not at all appropriate	Somewhat appropriate	Very appropriate

* 'Don't know' answers and missing values for each of these survey questions were interpolated using a mean score of the responder's answers to the other question(s)

§Total 'attitude' scores were calculated for each respondent. These were then dichotomised before further analysis with a score of seven or greater indicating a positive attitude, and a score of six or less a negative attitude

### Ethical approval

NHS ethical and Trust approvals were secured from Suffolk Local Research Ethics Committee (25^th ^May 2007; Ref: 07/Q0102/45), and Birmingham and Solihull PCT Consortium (Ref: 1087).

## Results

**Figure 1:**
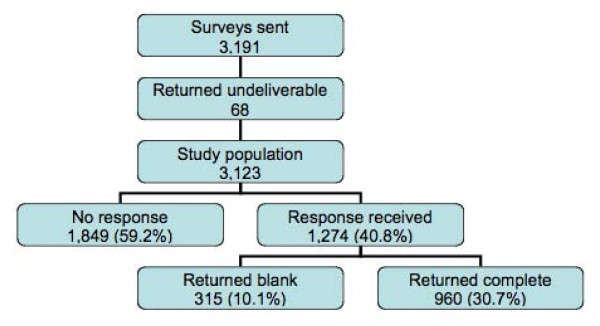
**Consort diagram detailing surveys mailed and returned**.

Of 3,191 surveys distributed, 68 (2.1%) were returned as 'undeliverable', and 315 (9.9%) were returned blank, indicating a desire not to receive a reminder. A further 1,849 recipients (57.9%) did not respond to either the initial mailing or the reminder, giving a total of 960 usable responses (response rate 30.7%), see Figure 1.

Some statistically significant differences between the GP practice characteristics of responders and non-responders were found. GPs in practices located in less deprived areas were more likely to respond; those from more deprived areas were under-represented (X^2 ^= 10.3; p = 0.016). Similarly, those from practices with larger numbers of GPs were significantly more likely to respond than those in single-handed practices (X^2 ^= 16.2; p = 0.003), and GPs working in practices with a greater proportion of patients in the white British ethnic group were over-represented in survey responses received (X^2 ^= 14.1; p = 0.007). However, when practices were aggregated by stratification factors (e.g. small/deprived, large/affluent), no statistically significant differences in response rates were observed between the groups (Table 2).

**Table 2:** Practice characteristics of survey responders vs. non-responders

Characteristic	Responders (%)	Non-responders (%)	All (%)	Significance
*Randomisation/stratification group*				
Small/Deprived	217 (22.6)	572 (26.4)	789 (25.3)	X^2 ^= 5.5 p = 0.141
Small/Affluent	253 (26.4)	531 (24.5)	784 (25.1)	
Large/Deprived	240 (25.0)	531 (24.5)	771 (24.7)	
Large/Affluent	250 (26.0)	529 (24.5)	779 (24.9)	
				
*IMD quartile*				
Quartile 1 (least deprived)	227 (23.6)	463 (21.4)	690 (22.1)	X^2 ^= 10.3 p = 0.016
Quartile 2	276 (28.8)	597 (27.6)	873 (28.0)	
Quartile 3	234 (24.4)	483 (22.3)	717 (23.0)	
Quartile 4 (most deprived)	223 (23.2)	620 (28.7)	843 (27.0)	
				
*GP count*				
Single handed	68 (7.1)	243 (11.2)	311 (10.0)	X^2 ^= 16.2 p = 0.003
2 to 4 GPs	495 (51.6)	1,056 (48.8)	1,551 (49.7)	
5 to 7 GPs	249 (25.9)	524 (24.2)	773 (24.8)	
8 to 10 GPs	115 (12.0)	239 (11.0)	354 (11.3)	
11 or more GPs	33 (3.4)	101 (4.7)	134 (4.3)	
				
*White British %*				
Less than 20	1 (0.1)	18 (0.8)	19 (0.6)	X^2 ^= 14.1 p = 0.007
20 to 39	29 (3.0)	89 (4.1)	118 (3.8)	
40 to 59	55 (5.7)	174 (8.1)	229 (7.3)	
60 to 79	106 (11.0)	229 (10.6)	335 (10.7)	
80 to 100	769 (80.1)	1,651 (76.4)	2,420 (77.5)	
Not known	0 (0.0)	2 (0.1)	2 (0.1)	
				
*Practice setting*				
Hamlet	5 (0.5)	12 (0.5)	17 (0.6)	X^2 ^= 12.5 p = 0.131
Village	62 (6.4)	96 (4.3)	157 (5.0)	
Town and fringe	142 (14.8)	306 (13.7)	436 (14.0)	
Urban	751 (78.2)	1,814 (81.3)	2,510 (80.4)	
Not known	0 (0.0)	3 (0.1)	3 (0.1)	

### Characteristics of respondents

The majority of survey respondents were White British (n = 719; 74.9%), with those considering themselves to be Asian/Asian British constituting 15.6% of responses received (n = 150). Most respondents had no personal experience of CRC screening, but 98 individuals (10.2%) had been screened in the past for CRC with one or more of the four available CRC screening modalities (FOBt, flexible sigmoidoscopy, colonoscopy or double contrast barium enema). Just over a quarter of respondents had some form of medical school affiliation (n = 259; 27.0%). In terms of GP practice attributes, GPs from mid-sized practices constituted the majority of respondents: 51.6% (n = 495) worked in practices with between two and four GPs, and a further 249 respondents (25.9%) were from practices with between five and seven GPs. Most respondents were partners or principals in their practice (87.1%; n = 836). Those in single handed practices comprised 7.1% of responses received (n = 68). Responses were split fairly evenly on the basis of deprivation quartile and practice list size (Table 3).

**Table 3:** Mean FOBt attitude scores, and OR indicating association between practice and socio-demographic characteristics of respondents with positive attitudes towards FOBt

Characteristic	Number of respondents (%) *	Mean FOBt attitude score §	Bivariate OR (95% CI)	Significance
**Practice Attributes**				
*Practice list size*				
Less than 3,800	200 (20.8)	6.0	1.1 (0.7 to 1.6)	p = 0.792
3,800 to 5,499	187 (19.5)	5.9	0.9 (0.6 to 1.4)	p = 0.742
5,500 to 7,999	215 (22.4)	5.9	0.9 (0.6 to 1.3)	p = 0.525
8,000 to 10,889	177 (18.4)	5.9	1.0 (0.6 to 1.5)	p = 0.940
10,890 or more	178 (18.5)	5.9	Reference	
*% of practice patients over 50*				
Less than 25	96 (10.0)	6.3	Reference	
25 to 49	530 (55.2)	5.9	0.7 (0.5 to 1.1)	p = 0.118
50 to 74	245 (25.5)	5.9	0.6 (0.4 to 1.0)	p = 0.053
75 to 100	3 (0.3)	6.0	0.7 (0.1 to 8.0)	p = 0.774
*Practice IMD quartile*				
Quartile 1 (least deprived)	227 (23.6)	5.9	0.7 (0.5 to 1.1)	p = 0.107
Quartile 2	276 (28.8)	5.8	0.6 (0.4 to 0.9)	p = 0.012
Quartile 3	234 (24.3)	5.9	0.7 (0.5 to 0.9)	p = 0.032
Quartile 4 (most deprived)	223 (23.2)	6.1	Reference	
*Practice GP count*				
Single handed	68 (7.1)	6.2	1.3 (0.5 to 3.0)	p = 0.603
2 to 4 GPs	495 (51.6)	6.0	0.9 (0.4 to 1.8)	p = 0.733
5 to 7 GPs	249 (25.9)	5.8	0.7 (0.3 to 1.5)	p = 0.354
8 to 10 GPs	115 (12.0)	5.9	0.9 (0.4 to 2.0)	p = 0.794
11 or more GPs	33 (3.4)	6.0	Reference	
				
**Socio-demographic characteristics**				
*Ethnic group*				
White British	719 (74.9)	5.9	Reference	
Asian/Asian British	150 (15.6)	6.3	1.9 (1.3 to 2.7)	p = 0.001
Other	88 (9.2)	5.9	1.3 (0.8 to 2.0)	p = 0.334
*Medical school affiliation*				
Yes	259 (27.0)	6.0	1.3 (1.0 to 1.8)	p = 0.094
No	621 (64.7)	5.9	Reference	
*Personal experience of CRC screening*				
Yes	98 (10.2)	6.4	1.6 (1.1 to 2.4)	p = 0.044
No	836 (87.1)	5.9	Reference	
All respondents	960 (100.0)	5.9		

* Percentages may not total 100 due to missing responses

§Minimum possible score = 3; maximum = 9: for calculation of Odds Ratios, scores were dichotomised, (scores of 6 or lower indicative of a negative attitude; 7 or higher showing a positive attitude). Reported OR indicate association between socio-demographic/practice characteristics and positive attitudes towards faecal occult blood testing

### Attitudes towards cancer screening

Established national cancer screening programmes were considered by the majority of respondents to be effective in reducing mortality in average-risk patients, with 95.1% of GPs (n = 893) perceiving cervical cancer screening to be either 'very' or 'somewhat' effective, and 96.7% (n = 908) believing the same of mammography. This compared with 77.7% of respondents (n = 729) who considered FOBt as similarly effective. FOBt was recommended to patients by GPs far less frequently than either cervical smears or mammography: only 11% of respondents (n = 105) stated that they recommended FOBt 'almost always' when appropriate for asymptomatic average-risk patients, compared with 96.5% (n = 926) recommending cervical screening and 87.2% (n = 837) who routinely endorse mammography.

### Attitudes towards colorectal cancer screening

Respondents were asked whether they considered each of the four principal CRC screening modalities to be 'very', 'somewhat', or 'not at all' appropriate for population based CRC screening. Despite colorectal cancer screening typically being recommended less frequently than other cancer screening tests, FOBt was considered by the majority of survey respondents to be an appropriate test. FOBt was considered 'very appropriate' by 50.1% of GPs (n = 481), compared with 11.8% (n = 113) believing the same of flexible sigmoidoscopy; 12.8% (n = 123) endorsing colonoscopy, and only 3.1% (n = 27) perceiving the double contrast barium enema to be a 'very appropriate' screening test (Table 4).

**Table 4:** Respondents' perceived appropriateness of CRC screening tests for population based CRC screening

	Faecal Occult Blood Test (%)	Flexible Sigmoidoscopy (%)	Colonoscopy (%)	Double Contrast Barium Enema (%)
Very appropriate	481 (50.1)	113 (11.8)	123 (12.8)	27 (3.1)
Somewhat appropriate	382 (39.8)	403 (42.0)	255 (26.6)	181 (20.7)
Not at all appropriate	79 (8.2)	405 (42.2)	546 (56.9)	665 (76.2)
Missing responses	18 (1.9)	39 (4.1)	36 (3.8)	87 (9.1)
Total (%)	960 (100.0)	960 (100.0)	960 (100.0)	960 (100.0)

FOBt was considered an inappropriate screening test by 8.2% of GPs (n = 79), compared with 42.2% (n = 405) agreeing that flexible sigmoidoscopy was inappropriate; 56.9% (n = 546) believing the same of colonoscopy, and 76.2% (n = 665) finding double contrast barium enema 'not at all appropriate'.

Of those who responded to the question, 31.4% of GPs (n = 72) agreed with the current recommended starting age for FOBt screening (60 years old). 148 GPs (64.6%) recommended beginning screening at a younger age, with only 3.9% of those responding (n = 9) believing that screening should begin at a later age than 60. The majority of respondents advocated less frequent testing than is currently recommended by the NHSBCSP. Of those who responded, 22.5% (n = 43) agreed with the current recommendation of biennial screening. 13.1% (n = 25) believed that people should be screened more frequently, and 64.4% (n = 123) advocated less frequent screening. The preferred option was screening every three years (37.2%; n = 71). There was little consistency in the reporting of a recommended stopping age for CRC screening in the asymptomatic average-risk population.

#### Factors influencing recommendation of CRC screening

Respondents were asked about the factors which influence whether or not they typically recommend CRC screening to their asymptomatic average-risk patients. Most influential were evidence published in the medical literature (569 GPs citing this as 'very' influential; 62.6%), and national policy relating to screening (n = 541; 63.6%). Amongst the least influential factors were screening uptake rates, with only 18.8% of GPs considering these to be very influential (n = 165), (Table 5).

**Table 5:** Factors influencing GP recommendation of CRC screening

Factor	Very influential (%)	Somewhat influential (%)	Not influential (%)	Total (%)
Clinical evidence in medical literature	569 (62.6)	306 (33.7)	34 (3.7)	909 (94.7)
Screening uptake rates	165 (18.8)	456 (51.9)	257 (29.3)	878 (91.5)
Continuing education/conferences/meetings	320 (35.6)	492 (54.7)	88 (9.8)	900 (93.8)
Primary Care Trust (PCT) policy	367 (40.7)	412 (45.7)	123 (13.6)	902 (94.0)
National policy	541 (63.6)	266 (31.3)	43 (5.1)	850 (88.5)

#### Perceived barriers to colorectal cancer screening

Respondents were asked about a series of patient and system related barriers to CRC screening, indicating whether they considered each to be a major, minor, or insignificant barrier. GP responses are shown in Table 6.

**Table 6:** Perceived barriers to colorectal cancer screening

Barrier	Not a barrier (%)	Minor barrier (%)	Major barrier (%)	Total (%)
**Patient related**				
Patient fear of finding cancer	97 (10.4)	612 (65.6)	224 (24.0)	933 (97.2)
Patient believes screening ineffective	262 (28.3)	489 (52.8)	175 (18.9)	926 (96.5)
Patient embarrassment/anxiety	57 (6.1)	410 (44.0)	465 (49.9)	932 (97.1)
Patient unaware of screening	57 (6.1)	268 (29.0)	599 (64.8)	924 (96.3)
Patient does not perceive CRC as a serious threat	246 (26.7)	437 (47.5)	237 (25.8)	920 (95.8)
**System related**				
Screening costs too much	116 (12.7)	355 (38.8)	444 (48.5)	915 (95.3)
GPs do not actively recommend screening	145 (15.8)	403 (44.0)	367 (40.1)	915 (95.3)
Shortage of trained providers to conduct screening	67 (7.4)	314 (34.8)	521 (57.8)	902 (94.0)
Shortage of trained providers to investigate positive FOBt	88 (9.9)	311 (34.9)	492 (55.2)	891 (92.8)

Both patient and system related barriers were seen by GPs as being influential, to varying degrees. Amongst patient related barriers, a lack of patient awareness was cited most frequently as a major obstacle to screening uptake, with 599 respondents (64.8%) believing this to be the case. Similarly important was perceived patient embarrassment and anxiety (n = 465; 49.9%). The patient barriers seen as the least problematic were a lack of patient belief in the screening effectiveness, and patient belief that colorectal cancer does not constitute a serious threat to health (n = 262; 28.3% and n = 246; 26.7% respectively).

All four of the system related barriers outlined in the survey were considered as major obstacles to CRC screening by around half of those who responded. In particular, the lack of trained healthcare providers to conduct screening, and the shortage of healthcare providers trained adequately to investigate positive FOB tests (n = 521; 57.8% and n = 492; 55.2% respectively). Analysis of respondent characteristics did not show any statistically significant differences between groups, according to socio-demographic or practice attributes, suggesting that the barriers to colorectal cancer screening were perceived as similarly influential regardless of the characteristics of individual GPs who responded.

#### Attitudes towards screening delivery

Respondents were in general strongly in favour of population-based CRC screening being organised centrally, as is the case in the NHSBCSP, rather than delivered directly by GPs, with 78.7% (n = 755) agreeing that centrally organised screening was appropriate. Similarly, despite a recognition by the majority of respondents that CRC screening could be effectively performed by trained nurse practitioners (77.5%; n = 744), 62.1% of GPs (n = 596) believed that CRC screening could be effectively performed by patients themselves, using home testing kits.

### Attitudes towards FOBt

Respondent attitudes towards FOBt were assessed through the calculation of an attitude score. The mean score for all respondents (n = 960) was 5.9. Only 43 respondents (4.5%) scored the maximum of nine (indicating a very positive attitude), and 6.8% (n = 65) scored the minimum of three (very negative attitude). Mean scores were calculated according to both practice attributes and socio-demographic characteristics of respondents, and remained very similar across all groups and sub-groups (Table 3). Binary logistic regression was used to calculate bivariate Odds Ratios evaluating the association of socio-demographic and practice attributes with FOBt attitude scores. No significant associations were found between FOBt attitude and practice list size; percentage of registered patients aged over 50; the number of GPs working in a practice, or whether or not a respondent indicated an affiliation with a medical school, although those with such an affiliation (n = 259; 27.0%) were more likely to have a positive attitude towards FOBt than those without (bivariate OR: 1.3; CI 1.0 to 1.8).

Personal experience of CRC screening was found to be significantly associated with a positive attitude towards FOBt, with respondents who had undergone CRC screening in the past (regardless of modality) more likely to have a positive attitude than those who had not (bivariate OR: 1.6; CI 1.1 to 2.4). Similarly, respondent ethnicity was found to have a significant association with a positive attitude towards FOBt. Those considering themselves Asian/Asian British were more likely than White British respondents to have a positive attitude (bivariate OR: 1.9; CI 1.3 to 2.7). A significant association was also found between practice deprivation quartile and FOBt attitude. Survey respondents from less deprived quartiles were *less *likely to have a positive attitude towards CRC screening with FOBt than those from the most deprived quartile - those in quartile two were nearly half as likely as those in quartile four to accept FOBt as beneficial (bivariate OR: 0.6; CI 0.4 to 0.9).

It might be expected that survey respondents from non-White British ethnic groups may be disproportionately concentrated in socio-economically deprived areas. However, even after controlling for the possible confounding influence of practice deprivation quartile on FOBt attitude by ethnic group, the significance of the observed associations remained, although they were less pronounced (multivariate OR for ethnic group: 1.8; CI 1.2 to 2.6; p = 0.002; multivariate OR for deprivation quartile two: 0.7; CI 0.5 to 0.9; p = 0.039).

## Discussion

Despite the recent establishment of the NHS Bowel Cancer Screening Programme, and the consequently limited direct engagement of primary care in administering screening for colorectal cancer, there will inevitably be ongoing demands on GPs to provide information and advice about CRC screening. These demands are likely to come from both patients within the age range covered by the NHSBCSP, due to the national advertising of the programme, and from those outside of the target age groups, prompted by commercial companies focusing on these populations [27]. Research has found that GPs can influence their patients in the decision to have CRC screening [28,29], and that GP recommendations can be particularly influential in prompting long term screening compliance [30]. Therefore, the positive engagement of GPs with CRC screening (and with FOBt in particular), is required if screening uptake rates are to reach acceptable levels, and the projected CRC mortality reductions observed in randomised controlled trials of FOBt screening are to be achieved in practice [30].

The findings from this survey raise a number of issues. CRC screening in general, and FOBt in particular, are typically perceived as less effective in their potential to reduce mortality amongst the appropriate target age groups than better established cancer screening tests such as cervical smears and mammography. This has important consequences for GP engagement with CRC screening (and for the uptake of population-based screening), as research has found the perceived efficacy of different screening options to be a clear determinant of clinical practice [21,22], despite comparable evidence of effectiveness from randomised controlled trials [31]. A number of patient and system-related barriers to screening uptake were also identified by respondents. However, the majority of GPs supported centrally organised delivery of CRC screening, as well as believing that population-based screening could be effectively performed by patients using home testing kits. This broad endorsement of the screening approach followed in the NHSBCSP suggests that GPs are largely supportive of the manner in which the programme is delivered, which may contribute positively to their likelihood of recommending FOBt to their patients.

Nevertheless, the factors affecting GP attitudes towards, and recommendation of, FOBt remain unclear. In line with other research evidence, we found few socio-demographic or GP practice attributes to be statistically significant determinants of attitudes towards CRC screening [22-24]. The inability of GP practice attributes in particular to account for observed attitudes suggests that we may need to consider the role of other, cross-cutting psychosocial, cultural or educational factors in explaining GP attitudes towards FOBt and their potential role in facilitating patient uptake of CRC screening. These cross-cutting factors may not be easily reducible to pre-defined demographic and associated characteristics. It may be the case that the greatest potential gains in increasing uptake of population-based CRC screening lie in better understanding and addressing patient-related factors in addition to those relating to healthcare professionals [32,33].

Finally, this survey found that GPs in non-White ethnic groups were *more *likely to have a positive attitude towards FOBt screening, and were consequently more likely to recommend it to their patients than those in the White British ethnic group. This runs counter to evidence (although much of it US based), that healthcare professionals belonging to certain ethnic minority groups are *less *likely to recommend bowel cancer screening to their patients, particularly where their patients are from similar ethnic backgrounds. This association was noted in work on screening uptake rates in the UK colorectal cancer screening pilot [14]. Here, CRC screening uptake rates were lower for individuals registered with an Asian GP, especially when the GP was of the Muslim faith. Uptake rates were similarly found to be lower for patients registered with single handed GP practices, and in those located in more deprived areas [34]. Our findings in relation to the associations between ethnicity and deprivation in determining GP attitudes to CRC screening using FOBt may be evidence of a disjuncture between attitudes, subsequent GP behaviour, and crucially, patient compliance with CRC screening which needs to be explored further through both qualitative and quantitative research.

## Limitations

There were some limitations to this study. First, the survey asked GPs about their attitudes towards CRC screening using FOBt and whether or not they typically recommend it to their patients. As previously discussed, we do not know if the stated attitudes conform to actual clinical practice, although respondents were assured of the confidentiality of their responses, thereby minimising the likelihood of false reporting. Nevertheless, in the absence of tangible measurements of GP practice, the degree to which *attitudes *translate into *behaviours *and the mechanisms by which this may occur remains unclear. Similarly, despite research evidence that GP recommendations can have beneficial impacts on patient compliance with CRC cancer screening, we do not know the extent to which the attitudes and perceptions expressed by our respondents have such effects on patient uptake of screening in this context. There is a clear need for research which undertakes assessment of GP attitudes alongside investigation into actual patient uptake of CRC screening.

A further limitation is the non-response bias observed with regard to the socio-demographic and GP practice attributes of survey respondents and non-respondents. With respondents from less deprived areas over-represented in the sample in comparison to those from more deprived locations, the observed association between deprivation and GP attitudes towards FOBt screening may be overstated. This may also have had an effect on the association seen between GP ethnic group and FOBt attitudes, since deprivation and ethnicity are known to be closely correlated. The findings outlined here must therefore be interpreted with caution.

Finally, the survey response rate (30.7%; n = 960) was relatively low in comparison to other postal surveys of healthcare professionals [35,36]. The reasons for this are unknown, but it is possible that only GPs motivated by, or interested in CRC screening responded to the survey, and that the observed positive attitudes towards FOBt outlined in the current study are over-represented. Nevertheless, the number of survey responses received constitutes a larger sample size than other surveys of GP attitudes towards CRC screening [21,22,28]. Many of these studies were conducted internationally, in healthcare settings different from that of the UK, whereas the current research reports findings directly relevant to the UK context.

## Conclusions

Colorectal cancer screening presents significant challenges for primary care, and further evidence is needed about the ways in which the delivery and uptake of screening programmes can be improved. The success of population-based screening for CRC will be determined to a large extent by GP attitudes, beliefs and support, particularly with regard to faecal occult blood testing. Previous research has implied that South Asian GPs are more likely to have negative attitudes towards FOBt. However, our research suggests that this is not a group that requires targeted interventions in order to increase their support for the NHSBCSP. Of the available CRC screening tests, GPs perceived FOBt to be the most appropriate for population-based screening. Nevertheless, only 50.1% of GPs reported finding this test 'very appropriate', and a number of patient and system-related barriers to screening uptake were identified by respondents.

## Competing interests

The authors declare that they have no competing interests.

## Authors' contributions

Survey data collection and analysis was carried out by SC. The first draft of this article was composed by SD, and was revised critically by all authors. All authors have read and approved the final manuscript.

## Pre-publication history

The pre-publication history for this paper can be accessed here:

http://www.biomedcentral.com/1471-2296/11/20/prepub

## Supplementary Material

Additional file 1 **Survey instrument**. Survey instrument used to collect data on GP attitudes and practices regarding faecal occult blood testing for colorectal cancer. Click here for file
