# Supplementary material for: Colorectal cancer screening using the faecal occult blood test (FOBt): a survey of GP attitudes and practices in the UK
Source: BMC Fam Pract. 2010 Mar 9;11:20. doi: 10.1186/1471-2296-11-20 (PMC2841115; doi:10.1186/1471-2296-11-20)
Supplement: Additional file 1 — Survey instrument. Survey instrument used to collect data on GP attitudes and practices regarding faecal occult blood testing for colorectal cancer. [file 1471-2296-11-20-S1.DOC]

**
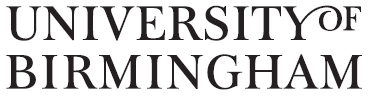
**

**Cancer Screening – a survey of General Practitioners’ attitudes and practices**

Cancer screening is defined in this survey as the routine, periodic use of a testing procedure intended to detect cancer or pre-cancerous lesions at an earlier stage than is possible through clinical detection or incidental discovery. Cancer screening is used in patients who display no signs or symptoms of possible cancer (i.e. pain, bleeding, palpable masses, etc.)

**Part 1 Cancer Screening**

| **1) How effective or ineffective do you believe the following screening procedures are in reducing cancer mortality in average-risk patients aged 50 years and older?** (Shade one circle on each line, as in the example) | | | | | | | | | | | | | | |
| --- | --- | --- | --- | --- | --- | --- | --- | --- | --- | --- | --- | --- | --- | --- |
|  |  | Very effective | | | | | Somewhat effective | | | | Not effective | | | Don’t know |
| **Example** of how to complete this questionnaire | | | |  | | | **●** | | | |  | | |  |
| a | Cervical smear |  | | | | |  | | | |  | | |  |
| b | Mammography |  | | | | |  | | | |  | | |  |
| c | Prostate specific antigen |  | | | | |  | | | |  | | |  |
| d | Faecal occult blood test |  | | | | |  | | | |  | | |  |
|  |  |  | | | | |  | | | |  | | |  |
| **2) In your practice, how often do you recommend the following cancer screening procedures for your asymptomatic average-risk patients of the appropriate age and gender?** (Shade one circle on each line). | | | | | | | | | | | | | | |
|  |  | | Almost always | | | | Sometimes | | | | Rarely | | | Never |
| a | Cervical smear | |  | | | |  | | | |  | | |  |
| b | Mammography | |  | | | |  | | | |  | | |  |
| c | Prostate specific antigen | |  | | | |  | | | |  | | |  |
| d | Faecal occult blood test | |  | | | |  | | | |  | | |  |
|  |  | |  | | | |  | | | |  | | |  |
| **3) Please complete the table below based on your recommendations to asymptomatic, average-risk patients for cancer screening. If you do not recommend a particular test, shade the circle in the last column**. | | | | | | | | | | | | | | |
|  |  | | Recommended starting age | | | Recommended frequency of testing | | | | | | Recommended stopping age | | I do not recommend |
| a | Cervical smear | |  | | yrs | every | | |  | yrs | |  | yrs |  |
| b | Mammography | |  | | yrs | every | | |  | yrs | |  | yrs |  |
| c | Prostate specific antigen | |  | | yrs | every | | |  | yrs | |  | yrs |  |
| d | Faecal occult blood test | |  | | yrs | every | | |  | yrs | |  | yrs |  |
| **Part 2 – Colorectal Cancer Screening** | | | | | | | | | | | | | | |
| **4) Which of the following screening tests do you think are appropriate for population based colorectal screening?** (Shade one circle on each line) | | | | | | | | | | | | | | |
|  |  | | Very appropriate | | | | | Somewhat appropriate | | | Not at all appropriate | | |  |
| a | FOB test | |  | | | | |  | | |  | | |  |
| b | Flexible sigmoidoscopy | |  | | | | |  | | |  | | |  |
| c | Colonoscopy | |  | | | | |  | | |  | | |  |
| d | Barium enema | |  | | | | |  | | |  | | |  |
| e | Other (Please specify) | |  | | | | |  | | |  | | |  |

| **5) What factors influence your recommendations for colorectal cancer screening?** (Shade one circle in each line) | | | | | | | | |
| --- | --- | --- | --- | --- | --- | --- | --- | --- |
|  |  | | | Very influential | | Somewhat influential | | Not influential |
| a | Clinical evidence published in the medical literature | | |  | |  | |  |
| b | Screening uptake rates | | |  | |  | |  |
| c | Continuing education/conferences/meetings | | |  | |  | |  |
| d | PCT policy | | |  | |  | |  |
| e | National policy | | |  | |  | |  |
| f | Other (Please specify) | |  | |  | |  | |
|  |  | | | | | | | |
| **6) In your opinion, how important are each of the following as barriers to colorectal cancer screening?** | | | | | | | | |
| Patient-Related Barriers (Shade one circle in each line) | | | | | | | | |
|  |  | | | Major barrier | | Minor barrier | | Not a barrier |
| a | Patient fear of finding cancer | | |  | |  | |  |
| b | Patient believes screening is not effective | | |  | |  | |  |
| c | Patient embarrassment or anxiety | | |  | |  | |  |
| d | Patient is unaware of screening | | |  | |  | |  |
| e | Patient does not perceive colorectal cancer as a serious health threat | | |  | |  | |  |
| f | Other (Please specify) |  | | |  | | | |
| System-Related Barriers | | | | | | | | |
| g | Screening costs too much | | |  | |  | |  |
| h | GPs do not actively recommend screening to their patients | | |  | |  | |  |
| i | Shortage of trained providers to conduct screening | | |  | |  | |  |
| j | Shortage of trained providers to investigate positive FOBTs | | |  | |  | |  |
| k | Other (Please specify) |  | | |  | | | |

| **PART 3. Faecal Occult Blood Testing (FOBT)**  *This section covers the use of FOBT to screen for colorectal cancer. Please respond based on how you actually practice even if this differs from how you would like to practice under ideal circumstances.* | | | | | | | | | | | | | | |
| --- | --- | --- | --- | --- | --- | --- | --- | --- | --- | --- | --- | --- | --- | --- |
| **7) For the majority of your patients, how would you conduct FOBT?** (Shade one circle) | | | | | | | | | | | | | | |
| a | Ask the patient to provide a stool sample for testing in the laboratory | | |  | | | b | | Complete a FOBT card in the surgery during a digital rectal exam | | | |  | |
| c | Give or mail patients a FOBT card to complete at home | | |  | | | d | | Other (Please specify) | |  | | | |
|  |  |  | | | |  | | | | | |  | |  |
| **8) To what extent would you recommend to your patients that they adhere to diet and drug restrictions such as abstaining from consumption of red meat or aspirin prior to completing the FOBT?** (Shade one circle) | | | | | | | | | | | | | | |
| a | I would recommend strict adherence to diet and drug restrictions | | | |  | | b | I would advise my patients to comply with diet and drug restrictions | | | | | |  |
| c | I would tell my patients not to worry about diet and drug restrictions | | | |  | | d | I would not discuss diet and drug restrictions with my patients | | | | | |  |
| e | Other (Please specify) | |  | | | | | | |  | | | |  |

| **9) Which of the following do you think you would recommend to an asymptomatic, average-risk patient as an initial follow-up step to a single positive FOBT?** (Shade circles for all that apply) | | | | | | | | | | | | | | | | | | | | | | | | | | | | | | | | | |
| --- | --- | --- | --- | --- | --- | --- | --- | --- | --- | --- | --- | --- | --- | --- | --- | --- | --- | --- | --- | --- | --- | --- | --- | --- | --- | --- | --- | --- | --- | --- | --- | --- | --- |
| a | Repeat FOBT | | | | | |  | | | | b | | Sigmoidoscopy | | | | | |  | | | | c | | Colonoscopy | | | |  | | | | |
| d | Double contrast barium enema | | | | | |  | | | | e | | Other (Please specify) | | | | | | | | |  | | | | | | | | | | | |
|  |  | | | |  | | | | | |  | | | | | | | | | | | | | | |  | | | |  | | | |
| **10) What is the capacity in your local area for procedures which you may require subsequent to a positive FOBT**. (Shade one circle in each line) | | | | | | | | | | | | | | | | | | | | | | | | | | | | | | | | | |
|  |  | | | | | | | | | More than enough to meet demand | | | | | Just about right | | | | | Inadequate for demand | | | | | | | | Don’t know | | | | Not applicable | |
| a | Flexible sigmoidoscopy | | | | | | | | | | |  | |  | | | | | | |  | | | | | |  | | | | | |  |
| b | Colonoscopy | | | | | | | | | | |  | |  | | | | | | |  | | | | | |  | | | | | |  |
| c | Double contrast barium enema | | | | | | | | | | |  | |  | | | | | | |  | | | | | |  | | | | | |  |
|  |  | | | |  | | | |  | | | | | | | | |  | | | | | | | |  | | | | |  | | |
| **11) Indicate whether you agree or disagree with the following statements about colorectal cancer screening**.  (Shade one circle in each line) | | | | | | | | | | | | | | | | | | | | | | | | | | | | | | | | | |
|  |  | | | | | | | | | | | Strongly agree | | Somewhat agree | | | | | | | Neither agree nor disagree | | | | | | Somewhat disagree | | | | | | Strongly disagree |
| a | Would be best delivered by GPs (similar to cervical cancer screening) | | | | | | | | | | |  | |  | | | | | | |  | | | | | |  | | | | | |  |
| b | Is best organised centrally (similar to breast cancer screening) | | | | | | | | | | |  | |  | | | | | | |  | | | | | |  | | | | | |  |
| c | Can be effectively performed by well-trained nurse practitioners | | | | | | | | | | |  | |  | | | | | | |  | | | | | |  | | | | | |  |
| d | Can be effectively performed by patients themselves using a home testing kit | | | | | | | | | | |  | |  | | | | | | |  | | | | | |  | | | | | |  |
|  |  | | | | | | |  | | |  | | | | | | | | | | | | | | |  | | | |  | | | |
| **12) During a typical month, how many times do you recommend or perform colorectal cancer screening with faecal occult blood testing for your asymptomatic, average-risk patients?** (Shade one circle) | | | | | | | | | | | | | | | | | | | | | | | | | | | | | | | | | |
|  | 0 |  | 1-10 |  | | 11-20 | | | | |  | | 21-40 | |  | More than 40 | | | | | | | |  | | Other (Please specify) | | | | | | | |
|  |  |  |  |  | |  | | | | |  | |  | |  | |  | | | | |  | | | |  | | | | | | | |

| **13) What brand of test do you use for screening for asymptomatic patients?** (Shade circles for all that apply) | | | | | | | | | |
| --- | --- | --- | --- | --- | --- | --- | --- | --- | --- |
| a | I don’t perform colorectal screening |  | b | Hemoccoult 11 |  | | c | Hemeselect |  |
| d | Hemoccoult Sensa |  | e | Colo-screen |  | | f | Hemoccult SP |  |
| g | Don’t know |  | h | Other (Please specify) | |  | | | |

| **14) What are your reasons for not recommending colorectal cancer screening with FOBT for asymptomatic average-risk patients?** (Shade circles for all that apply) | | | | | | | |
| --- | --- | --- | --- | --- | --- | --- | --- |
| a | Too many false negatives (inadequate sensitivity) | |  | b | Too many false positives (inadequate specificity) | |  |
| c | Too inconvenient for patients | |  | d | Providing a stool sample is not acceptable to patients | |  |
| e | Poor patient compliance | |  | f | Inadequate reimbursement | |  |
| g | Screening is not a good idea | |  | h | None of the above – I do recommend FOBT | |  |
| i | Other (Please specify) |  | | | |  | |

| **PART 4. You and your Practice**  *The questions in this final section will help us to better understand your medical practice.* | | | | | | | | | | | | | | | | | | | | | | | | | | | | | | | | | | | | | | | | |  |
| --- | --- | --- | --- | --- | --- | --- | --- | --- | --- | --- | --- | --- | --- | --- | --- | --- | --- | --- | --- | --- | --- | --- | --- | --- | --- | --- | --- | --- | --- | --- | --- | --- | --- | --- | --- | --- | --- | --- | --- | --- | --- |
| **15) Which of the following categories best describes your main practice arrangement (i.e. the practice setting where you spend the most hours per week)?** (Shade one circle) | | | | | | | | | | | | | | | | | | | | | | | | | | | | | | | | | | | | | | | | |  |
| a | Partner in a practice | | | | | | |  | | | | b | | Employee in a practice | | | | | | |  | | | | | c | | | Registrar | | | | | | | |  | | | |  |
| d | Employee of a hospital, clinic, or university practice | | | | | | |  | | | | e | | Other (Please specify) | | | | | | |  | | | | | | | | | | | | | | | | | | | |  |
|  |  | |  | | | | | | |  | | | | | | | | | | | | | | |  | | | | | | | | | | |  | | | | |  |
| **16) How many patients are registered on the practice list?** | | | | | | | | | | | | | | | | | | | |  | | | | | | | | | |  | | | | | | | | | | |  |
|  |  | |  | | | | | | |  | | | | | | | | | | | | | | |  | | | | | | | | | | |  | | | | |  |
| **17) Approximately what percentage of your patients are 50 years of age and older?** (Shade one circle) | | | | | | | | | | | | | | | | | | | | | | | | | | | | | | | | | | | | | | | | |  |
| a | Less than 25% | |  | b | | | | | 25-49% | | | |  | | | | c | 50-74% | | | | |  | | | | | | | d | | 75-100% | | | | | |  | | |  |
|  | | | | | | | | | | | | | | | | | | | | | | | | |  | | | | | | | | | | |  | | | | |  |
| **18) Do you as an individual have an affiliation with a medical school?** | | | | | | | | | | | | | | | | | | | | | | | Yes | | | |  | | | | No | | | |  | | | | | |  |
|  |  | |  | | | | | | |  | | | | | | | | | | | | | | |  | | | | | | | | | | |  | | | | |  |
| **19) Regarding your ethnic background, which group do you consider yourself to be in?** (Shade one circle) | | | | | | | | | | | | | | | | | | | | | | | | | | | | | | | | | | | | | | | |  | |
|  | White |  | | | | | | Black Caribbean | | | | | | | |  | | | Black African | | | | | | | |  | | | | | |  | | | | | | | | |
|  | Black British |  | | | | | | Indian | | | | | | | |  | | | Pakistani | | | | | | | |  | | | | | |  | | | | | | | | |
|  | Bangladeshi |  | | | | | | Chinese | | | | | | | |  | | | Other (Please specify) | | | | | | | | | | | |  | | | | | | | | | | |
|  |  | |  | | | |  | | | | | | | | | | | | | | | | |  | | | | | | | | | |  | | | | | | | |
| **20) Have you personally ever been screened for colorectal cancer?** (Shade circles for all that apply) | | | | | | | | | | | | | | | | | | | | | | | | | | | | | | | | | | | | | | | | | |
| a | Yes, with FOBT | | | | |  | | | b | | Yes, with sigmoidoscopy | | | | | | | | | | |  | | c | | | | Yes, with colonoscopy | | | | | | | | | | |  | | |
| d | Yes, with double contrast barium enema | | | | |  | | | e | | No, I have not been screened | | | | | | | | | | |  | |  | | | |  | | | | | | | | | | |  | | |
|  |  | | | |  | | | | | | | | | |  | | | | | | | | |  | | | | | | | | | |  | | | | | | | |
| **21) Is there anything else you would like to tell us about colorectal cancer screening in your practice or in general?** | | | | | | | | | | | | | | | | | | | | | | | | | | | | | | | | | | | | | | | | | |
|  | | | | | | | | | | | | | | | | | | | | | | | | | | | | | | | | | | | | | | | | | |
| **Thank you very much. We greatly appreciate your participation. Study results will help us to better understand the emerging and challenging area of colorectal cancer screening. Please return your completed survey in the enclosed FREEPOST envelope.** | | | | | | | | | | | | | | | | | | | | | | | | | | | | | | | | | | | | | | | | | |

If you have any questions about the study, please contact:

Sue Wilson, Department of Primary Care and General Practice, Primary Care Clinical Sciences Building, University of Birmingham, Edgbaston, Birmingham. B15 2TT United Kingdom. Telephone: 0121 414 7397

**All information provided will be held in accordance with the Data Protection Act 1998**

*123*

123
